# 3D macroporous electrode and high-performance in lithium-ion batteries using SnO_2_ coated on Cu foam

**DOI:** 10.1038/srep18626

**Published:** 2016-01-04

**Authors:** Ji Hyun Um, Myounggeun Choi, Hyeji Park, Yong-Hun Cho, David C. Dunand, Heeman Choe, Yung-Eun Sung

**Affiliations:** 1 School of Chemical and Biological Engineering, Seoul National University, Seoul 151-742, Republic of Korea; 2 Center for Nanoparticle Research, Institute for Basic Science (IBS), Seoul 151-742, Republic of Korea; 3 School of Advanced Materials Engineering, Kookmin University, Seoul 136-702, Republic of Korea; 4 Department of Chemical Engineering, Kangwon National University, Samcheok 245-711, Republic of Korea; 5 Department of Materials Science and Engineering, Northwestern University, Evanston, IL 60208, USA; 6 Cellmotive Co. Ltd., #518, Eng. Bldg., Kookmin University, Seoul 136-702, Republic of Korea

## Abstract

A three-dimensional porous architecture makes an attractive electrode structure, as it has an intrinsic structural integrity and an ability to buffer stress in lithium-ion batteries caused by the large volume changes in high-capacity anode materials during cycling. Here we report the first demonstration of a SnO_2_-coated macroporous Cu foam anode by employing a facile and scalable combination of directional freeze-casting and sol-gel coating processes. The three-dimensional interconnected anode is composed of aligned microscale channels separated by SnO_2_-coated Cu walls and much finer micrometer pores, adding to surface area and providing space for volume expansion of SnO_2_ coating layer. With this anode, we achieve a high reversible capacity of 750 mAh g^−1^ at current rate of 0.5 C after 50 cycles and an excellent rate capability of 590 mAh g^−1^ at 2 C, which is close to the best performance of Sn-based nanoscale material so far.

Developing technologies for portable electronic devices, electric vehicles, and grid-scale energy storage applications demand for high-performance lithium-ion batteries (LIBs) with high energy and high power densities and good cycling stabilities^1^,^2^. Tin dioxide (SnO_2_), as a promising alternative to currently used graphitic anode for next generation LIBs, has received much attention because of its high theoretical capacity of 781 mAh g^−1^, which is 2 times higher than that of the conventional graphitic anode (372 mAh g^−1^)^3^,^4^. However, practical application of the SnO_2_-based anode has been hindered by the inherently severe volume changes (up to 300%) during the large amounts of Li ion insertion and extraction; this can cause a pulverization of active material and a loss of electrical contact, and eventually resulting in poor capacity retention^3^,^4^,^5^. To resolve this issue, several strategies have been proposed, such as nanoscale electrodes^6^,^7^,^8^, electrodes hybridized with carbon or polymers^9^,^10^,^11^,^12^, and electrodes designed for unique architectures^13^,^14^,^15^,^16^.

A three-dimensional (3D) porous metallic architecture should have several important benefits including the following: (i) facile access of electrolyte to the electrode surface, (ii) facilitated charge transfer across the interface between electrode and electrolyte, (iii) relieved stress on the pulverization of active material by providing void spaces to absorb the large volume changes, additionally, (iv) high electron pathways in the electrode assembly^17^,^18^,^19^. Various fabrications about the 3D metallic scaffold have been explored such as inverse opal structure^18^,^20^, stainless steel mesh^21^, foams^22^,^23^,^24^, long chains of particles^25^, and fibers^26^ or wires^27^ assembly. Furthermore, a dual pore-size and pore-shape distribution architecture, interdigitated highly porous metallic scaffold, can enhance the volume density of active material by increasing surface area of the 3D scaffold as a template for deposited active material or as an active material itself^18^,^28^. A freeze-casting process is an easy, versatile, and promising method to prepare a highly interconnected microscale pore structure^29^,^30^. In this low-cost and scalable process, a solution is frozen and the frozen solvent is then removed by freeze-drying, forming a macroporous body structure as a replica of the solidified solvent structure^29^,^30^. As it is a physical process without chemical reactions, and as it uses ice crystals obtained from water or other forms of liquid template, freeze-casting generally results in pores at the surface of structure on the order of tens of micrometers in size concurrently with channel-like pores of several tens of micrometers in the structure.

We herein report a new SnO_2_ anode design concept, for the first time, based on a 3D macroporous Cu foam with a dual pore-size distribution by a directional freeze-casting technique. The Cu foam is utilized as both an anode current collector and a template for a SnO_2_ coating layer. The 3D macroporous Cu foam provides both continuous metallic struts to act as effective electron pathways and local void spaces to alleviate stress generated from large volume changes of SnO_2_ coating layer during cycling. This combination of properties in the electrode demonstrates a high reversible capacity, superior rate capability, and stable cycle retention with preserving its structural integrity.

## Results

### Material and structure design

**Figure 1:**
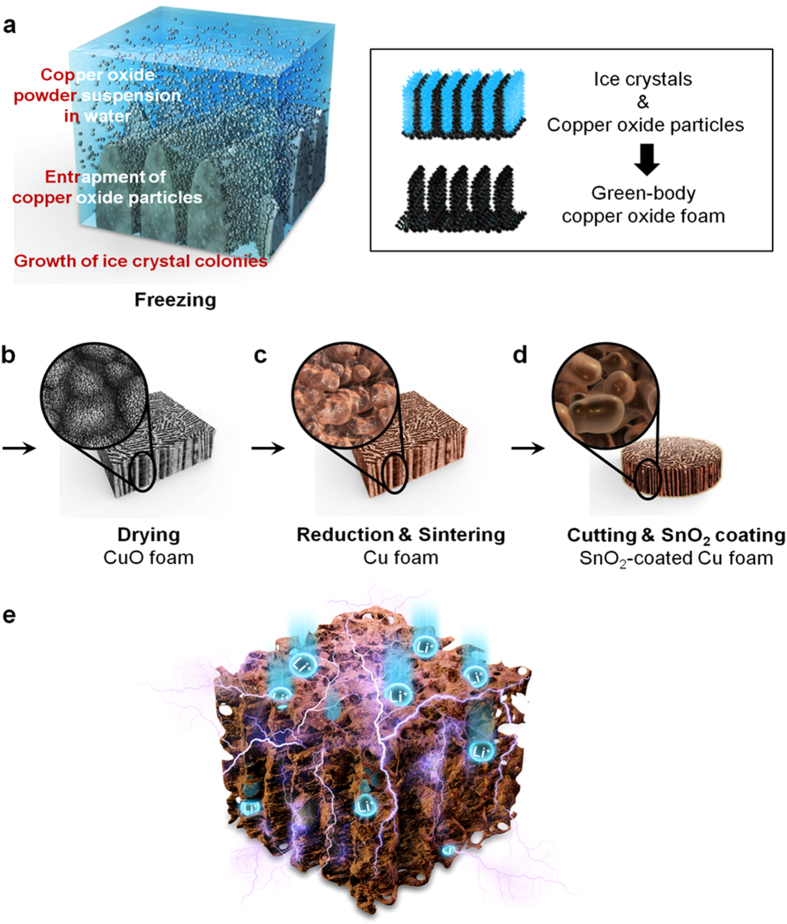
Electrode fabrication and architecture. (**a**–**d**) Schematic diagram of a fabrication process for SnO_2_-coated Cu foam electrode. (**a**) During directional freezing of CuO slurry, growth of vertical ice crystal colonies occurred simultaneously with entrapment of CuO particles between the ice crystals structure. A porous body structure was formed as a replica of the ice crystals (right box). (**b**) After drying of the ice, a porous layer-by-layer structure resulted, consisting of lightly-bound CuO particles. (**c**) The CuO foam was then sintered and reduced from dark green CuO to orange Cu in a hydrogen atmosphere. (**d**) A SnO_2_ sol-gel coating method was employed to fabricate a SnO_2_-coated Cu foam electrode. (**e**) Conceptual diagram of the SnO_2_-coated Cu foam with continuous metallic and porous structure enabling both effective electron and Li ion pathways and also stress alleviation in volume changes of SnO_2_ coating layer during cycling.

3D porous scaffold architecture is regarded as a smart electrode prototype for providing efficient Li ion and electron transport^17^,^18^,^19^. Recently, we have shown that commercially available Cu foam exhibits good electrochemical performance when used in anode for LIBs^24^. In this work, a freeze-casting process is employed to enhance the volumetric density of Cu scaffold. The freeze-casting technique was initially invented for use with porous ceramics and polymers because of their lower densities and the ease of suspension of particles in slurries. Since the first report on the use of biomedical materials obtained from collagen solutions in 1998^31^, the viability of freeze-casting for ceramic-based porous biomaterials has been widely investigated. In contrast, freeze-cast metals were not created until recently due to difficulties with particle settling in, and reactivity with, the solvent. Such foams were fabricated through the freeze-casting using titanium powder^32^, copper oxide powder which was reduced to copper^33^, and iron oxide powder^34^. Among the prepared (Ti, Cu, and Fe_2_O_3_) foams, the Cu foam can be regarded as the best scaffold used for a current collector because of the highest electrical conductivity. As a further step, SnO_2_ as an active material should be combined with the Cu scaffold current collector to react with Li ions. For advanced Sn-based materials, various synthesis methods have been developed such as sputtering, chemical vapor deposition, and electron beam deposition and so on. However, these methods have low throughput and require vacuum conditions, making economical scale-up difficult. One alternative approach is to use a sol-gel method, which has many advantages over the conventional methods using solid-state reactions. For instance, the sol-gel method can provide good stoichiometric control, homogeneous molecular mixing, an uniform particle size distribution^35^; in addition, it does not require a vacuum system and is thus scalable at low cost. We therefore chose to utilize a sol-gel method^36^ to apply a SnO_2_ coating to the Cu scaffold current collector. The fabrication processes of Cu foam via directional freeze-casting and SnO_2_ coating via sol-gel methods are schematically illustrated in Fig. 1a–d. One can thus fabricate a SnO_2_-coated Cu foam (hereafter, referred to as SnO_2_/Cu foam) anode by integrating four simple processes: freeze-casting, drying, reduction/sintering, and sol-gel coating, which can be linked into a single, continuous process that is able to scale up to very high throughput. The above processes result in the formation of a 3D continuous metallic and porous Cu foam covered with SnO_2_ coating layer inside, which is advantageous for both electron and Li ion transport and also volume changes of SnO_2_ coating layer during cycling (Fig. 1e).

### Fabrication of SnO_2_-coated Cu foam electrode

**Figure 2:**
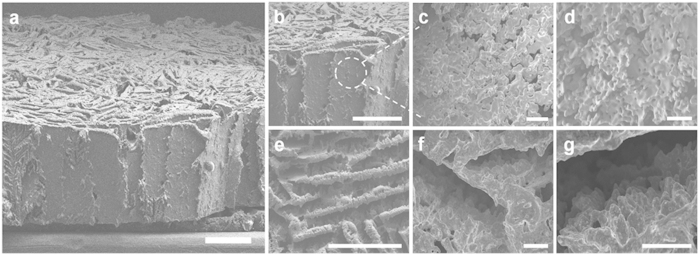
Freeze-cast Cu foam of macroporous layer-by-layer assembly. (**a**) SEM image of a freeze-cast Cu foam showing a vertically aligned Cu multilayer assembly. Scale bar, 400 μm. (**b–d**) Side view SEM images of the macroporous layer-by-layer Cu foam. (**b**) An enlarged portion of the Cu foam. Scale bar, 400 μm. (**c**) A porous Cu layer resulted from dendritic growth of ice crystals. Scale bar, 40 μm. (**d**) The Cu layer with interconnected microscale pores is also confirmed at higher magnification. Scale bar, 40 μm. (**e–g**) Top view SEM images of the macroporous layer-by-layer Cu foam. (**e**) A randomly oriented layered structure. Scale bar, 400 μm. (**f**) The development of dendrites in side view simultaneously observed from (**c,d**) in front view. Scale bar, 40 μm. (**g**) The dendritic-like morphology character is also confirmed at higher magnification. Scale bar, 40 μm.

FE-SEM images (Fig. 2a–g) show morphologies of an as-prepared Cu foam prior to application of the SnO_2_ coating. The Cu foam indicates various morphological characteristics upon comparing side views (Fig. 2b–d) with top views (Fig. 2e–g). Figure 2a shows a layered lamellar assembly of Cu walls or lamellae. The magnified surface morphologies on a lamella (Fig. 2c,d) as indicated by the white dotted circle in Fig. 2b show a high degree of roughness caused by dendritic-like morphologies of the ice crystals^30^, and confirm the presence of numerous small pores *ca.* 10 μm in diameter. The top images of Cu foam in Fig. 2a,b perpendicular to the ice front exhibit a randomly oriented layered structure. Although the lamella thicknesses and the intervals between lamellae vary considerably (Fig. 2e), they are estimated to be below 50 and 100 μm, respectively. Figure 2f,g display the development of dendrites in side view, which are also observed from Fig. 2c,d in front view. Moreover, the cross-sectional optical images of as-prepared Cu foam are shown in vertical and horizontal views (Supplementary Fig. S1a,b, respectively), revealing numerous, continuous, macroporous walls several tens of microns in width. It is also observed that lamellar bridges originating from overgrown dendrites eventually crossed the gaps between two adjacent lamellae^30^; an example is indicated by blue circles in Supplementary Fig. S1b. This observation suggests that the layer-by-layer macroporous structure can make connections continuously in the horizontal direction, resulting in a well-constructed three-dimensional Cu network structure. To evaluate the pore-size and distribution for as-prepared Cu foam, a mercury intrusion porosimetry (MIP) test was carried out. Because the MIP test could not detect the main channel-like pores of over 100 μm in diameter, those secondary pores on the order of a few tens of microns were detected. The major peak of the pore distribution is 14 μm, as seen in Supplementary Fig. S2, in qualitative agreement with the image in Fig. 2d; these pores is resulted from the secondary dendritic growth of ice crystals. In addition, the surface area of Cu foam via freeze-casting process is higher than that of commercially available Cu foam in previous our work^24^. The inset images of Supplementary Fig. S2 also show the cross-sectional area of Cu foam and its magnified surface morphology, confirming the existence of dendritic ice crystals.

**Figure 3:**
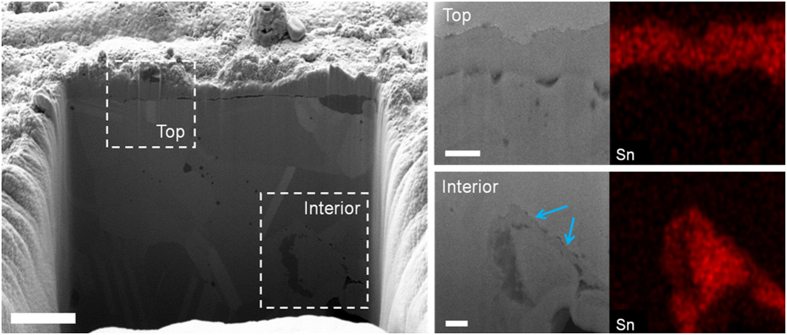
SnO_2_-coated Cu foam electrode. Cross-sectional SEM images of a SnO_2_/Cu foam electrode in its entirety and at top and interior regions as indicated by the white dotted rectangles with Sn element mapping. The blue arrows indicate interior SnO_2_ coating layer surrounding a secondary pore. Scale bars, 2 μm (entirety), 500 nm (top) and 500 nm (interior), respectively.

Figure 3 shows the cross-sectional images of SnO_2_/Cu foam taken by focused ion beam (FIB) milling and EDX mapping analyses, and confirms the presence of a SnO_2_ coating after sol-gel process (All elements including Sn are shown in Supplementary Fig. S3). The regions of interest, which are indicated by the white dotted rectangles in Fig. 3, are randomly selected for comparison of the top and interior areas of the SnO_2_/Cu foam. A SnO_2_ layer at the top is coated onto the Cu foam and estimated to be *ca.* 500 nm in thickness. Moreover, the presence of an interior SnO_2_ coating layer is clearly illustrated, as indicated by the blue arrows, surrounding a pore in the Cu foam arising from the dendrite formation on the lamella surface. The interior SnO_2_ coating layer is thinner than the top SnO_2_ coating layer. To confirm the presence of a SnO_2_ coating in the middle of 300 μm thick SnO_2_/Cu foam, cross-sectional SEM and EDX mapping images were taken at *ca.* 150 μm depth position. Supplementary Fig. S4a presents an entire cross-section of the SnO_2_/Cu foam mounted in polymer resin and polished according to standard metallographic procedures. The magnified surface on lamella in the SnO_2_/Cu foam interior (Supplementary Fig. S4b) indicates the border between the Cu wall and polymer resin at *ca.* 150 μm depth position. The lamella in SnO_2_/Cu foam interior is covered with a bright thin layer, implying the presence of Sn at *ca.* 150 μm depth position (Supplementary Fig. S4c,d). Supplementary Fig. S5 compares XRD patterns of the as-prepared Cu foam, SnO_2_/Cu foam and SnO_2_ powder directly obtained from sol-gel process and commercial SnO_2_ powder slurry pasted onto copper foil as control (hereafter, referred to as SnO_2_ SG and SnO_2_ NPs, respectively). The XRD pattern of as-prepared Cu foam identifies diffraction peaks for metallic copper (JCPDS 04-0836). The diffraction peaks observed in both SnO_2_/Cu foam and SnO_2_ NPs are assigned to either tetragonal rutile-structure SnO_2_ (JCPDS 41-1445) or Cu (JCPDS 04-0836), with no additional peaks corresponding to other phases such as SnO and Cu_2_O. Moreover, the oxidation stability of 3D Cu foam was examined in detail and discussed in Supplementary Fig. S5c.

### Electrochemical properties

To understand the electrochemical properties of SnO_2_/Cu foam electrode, cyclic voltammetry (CV) measurement was performed in the range of 0.01–2.0 V at a scan rate of 0.1 mV s^−1^ (Supplementary Fig. S6a). The three CV profiles of SnO_2_/Cu foam obtained from different sol concentration are in accordance with the electrochemical behavior of SnO_2_-based anode materials^37^,^38^. The normalized CV profiles display the reactive surface area of electrode, which can imply the highest porosity development of SnO_2_ coating layer from the 10 M of Sn(II) solution. The pH and/or viscosity resulted from the different sol concentration can affect the gelation environment and the development of porous gel^39^. In addition, the effect of sol concentration on LIBs performance was measured at current rate of 1 C (1 C = 781 mA g^−1^). The Li ion capacity is increased by increasing the Sn(II) solution as presented in Supplementary Fig. S6b, as a result, sol concentration was controlled at 10 M for subsequent experiments.

**Figure 4:**
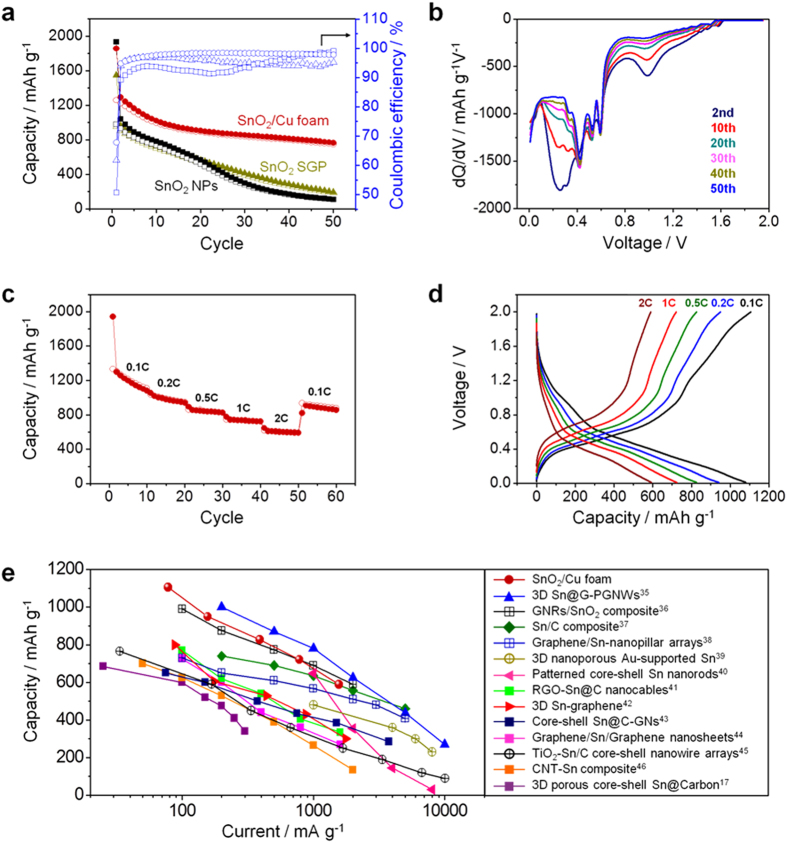
Electrochemical properties. (**a**) Cycle performance of SnO_2_/Cu foam, SnO_2_ SG, and SnO_2_ NPs at current rate of 0.5 C during 50 cycles and their coulombic efficiencies. (**b**) Differential capacity profiles of the SnO_2_/Cu foam differentiated from the discharge voltage profile after the selected cycles. (**c**) Rate performance of the SnO_2_/Cu foam at various C-rates from 0.1 C to 2 C. (**d**) Voltage profiles of the SnO_2_/Cu foam at the final discharge/charge processes in each current rate step. (**e**) Comparison of capacity at different current rates for the SnO_2_/Cu foam with other Sn-based anode materials reported in recent years. All the specific capacity and current rate are calculated based on the mass of SnO_2_.

The voltage profiles of SnO_2_/Cu foam, SnO_2_ SG, and SnO_2_ NPs during the first two cycles at 0.5 C are shown in Supplementary Fig. S7. All electrodes are similar in electrochemical pathway, corresponding to previous reports of the SnO_2_-based anode^37^,^38^. The SnO_2_/Cu foam delivers a capacity of 1856 mAh g^−1^ at the first discharge curve and a reversible capacity of 1258 mAh g^−1^. The discharge curve exhibits a plateau at ~0.9 V resulted from the formation of solid electrolyte interface (SEI) layer and the conversion reaction of SnO_2_ into Sn and Li_2_O. The second discharge and charge capacities of SnO_2_/Cu foam are 1292 mAh g^−1^ and 1224 mAh g^−1^, respectively. The enhanced capacity over the theoretical capacity of 781 mAh g^−1^ based on the conventional alloying reaction (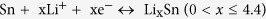) can be resulted from the irreversible capacity from electrolyte decomposition and the reversibility of conversion reaction (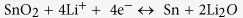). When the conversion reaction is totally reversible, the theoretical capacity of SnO_2_ increases up to 1494 mAh g^−1^. The second discharge curve differs from the first in that the voltage plateau disappeared, suggesting that the formation of SEI layer and Li_2_O occur mainly in the first cycle^37^,^38^. The cycling performance of SnO_2_/Cu foam, SnO_2_ SG, and SnO_2_ NPs at 0.5C was evaluated in Fig. 4a. Although the capacities of all electrodes degrade with similar decreasing rate during the first 10 cycles, the SnO_2_/Cu foam subsequently exhibits the superior capacity retention, in contrast to the severe capacity degradation almost 90% of both SnO_2_ SG and SnO_2_ NPs. Moreover, the capacity of SnO_2_/Cu foam after 50 cycles presents a relatively high capacity of 750 mAh g^−1^, which is better than all but one^40^ of Sn-based anode reported recently^22^,^41^,^42^,^43^,^44^,^45^,^46^,^47^,^48^,^49^,^50^,^51^. The coulombic efficiency of SnO_2_/Cu foam is ~68% at the first cycle, but is ~95% after the second cycle and maintains at ~98% after 50 cycles, showing good capacity retention. Considering the existence of CuO and Cu_2_O as discussed in Supplementary Fig. S5c and the possibility of their reactivity with lithium, the active material can be extended to sum of SnO_2_, CuO, and Cu_2_O. Therefore, the gravimetric performance of SnO_2_/Cu foam can decrease by maximum of 7.28% as shown in Supplementary Fig. S8. The electrochemical reactions of electrode during discharge and charge processes can be more clearly illustrated by the differential capacity profiles; that is the differential capacity *vs.* voltage plots. Fig. 4b indicates the differential capacity profiles of SnO_2_/Cu foam at the selected cycles. The distinct peaks between 0.7 V and 0.01 V after the second cycle correspond to the specific lithiation steps in forming Li_x_Sn^52^,^53^. The lithiation peaks of SnO_2_/Cu foam exhibit relatively stable behaviors after the 20th cycle, suggesting that the reversible Li ion insertion into SnO_2_/Cu foam can continue.

The rate capability of SnO_2_/Cu foam was evaluated by increasing the current rate from 0.1 C to 2 C stepwise and then decreasing back to 0.1 C, as shown in Fig. 4c. When cycled at 0.1 C, the initial charge capacity of 1330 mAh g^−1^ decreased gradually down to 1110 mAh g^−1^ by the 10th cycle. However, the capacity begins to show stable capacity retention and exhibits excellent cycling stability up to the 50th cycle, where the capacity is approximately 590 mAh g^−1^ at 2 C. After 50 cycles, when the current rate has been returned to 0.1 C, the capacity of SnO_2_/Cu foam has recovered considerably, showing a good rate capability. Figure 4d exhibits the final discharge and charge voltage profile in each current rate step, for the measurements presented in Fig. 4c. Even at the highest current rate of 2 C, lithiation is still observed as shown by a sloping voltage profile around 0.1 V, corresponding to the formation of Li_x_Sn^52^,^53^. In addition, the lithiation through forming Li_x_Sn even at 2 C is more clearly presented in Supplementary Fig. S9. Figure 4e compares the rate capabilities among a number of different Sn-based anode materials reported in recent years, which confirms that the superior rate capability of SnO_2_/Cu foam examined in this study is close to the best performance of Sn-based nanoscale material so far^40^.

## Discussion

**Figure 5:**
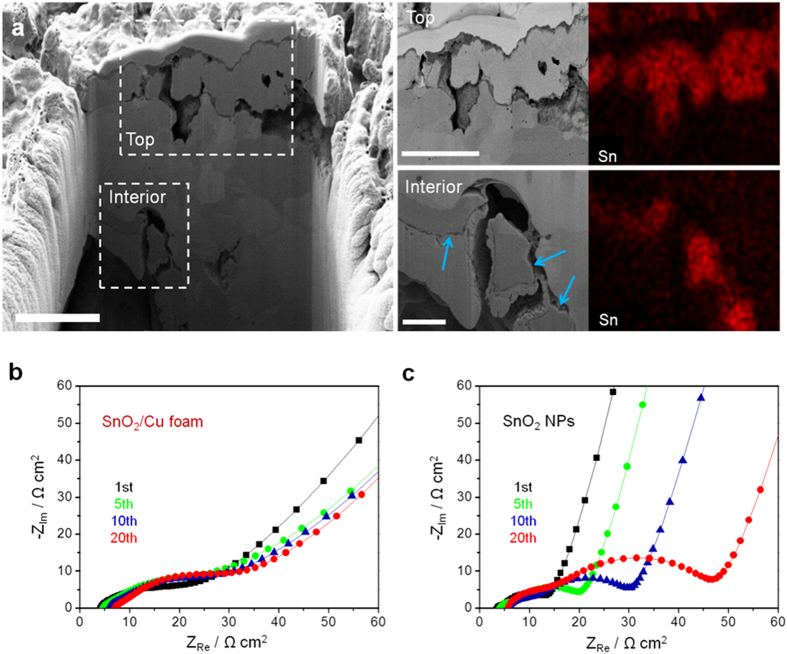
Morphology of SnO_2_/Cu foam electrode after 50 cycles and cell impedance test. (**a**) Cross-sectional SEM images of the SnO_2_/Cu foam electrode after 50 cycles at 1 C in its entirety and at top and interior regions as indicated by the white dotted rectangles with Sn element mapping. The blue arrows indicate a preserved interior SnO_2_ coating layer during cycling. Scale bars, 4 μm (entirety), 4 μm (top), and 1 μm (interior), respectively. (**b**) Cell impedance tests of the SnO_2_/Cu foam and SnO_2_ NPs after the selected cycles at 1 C.

There are following advantageous characteristics of such a 3D macroporous electrode design that imparts the SnO_2_-coated Cu foam with stable cycling performance and good rate capability: (i) intrinsic structural integrity of the 3D open network with interconnected pores and continuous metallic walls within the electrode enables faster transport of both Li ion and electron, (ii) presence of the smaller surface pores in the dual pore-size Cu scaffold creates a larger surface area and increases the contact area between active material and current collector, markedly decreasing the interfacial resistance, (iii) Cu foam with interconnected pores can relieve the stress caused by the large volume changes of coated SnO_2_ during cycling. In Supplementary Fig. S10a, the coin shape of SnO_2_/Cu foam electrode after cycling is maintained, but the side and top morphologies at high magnification are coarse and pulverized as shown in Supplementary Fig. S10b,c, respectively. To elucidate the excellent stabilizing effect of SnO_2_/Cu foam, the cross-section of SnO_2_ coating was examined using FIB milling and EDX mapping analyses after 50 cycles at 1C (Fig. 5a). All elements including Sn are shown in Supplementary Fig. S10d. The top SnO_2_ coating layer is significantly expanded to ~2 μm after 50 cycles compared to the initial top coating layer of 500 nm (Fig. 3). However, the interior SnO_2_ coating maintains a similar thin layer as indicated by the blue arrows, demonstrating that the interior coating layer is preserved without being pulverized by the significant volume changes from repeated Li ion insertion and extraction. In addition, electrochemical impedance spectroscopy (EIS) measurement of the SnO_2_/Cu foam and SnO_2_ NPs after completion of the different cycles at 1 C was conducted (Fig. 5b,c). There is no obvious impedance increase in SnO_2_/Cu foam, implying limited growth of the SEI layer, which is ascribed to the interior thin SnO_2_ coating layer in the Cu scaffold, whereas the impedance of SnO_2_ NPs corresponding to the SEI layer increases continuously. The stable SEI of SnO_2_/Cu foam until 50 cycles is confirmed in Supplementary Fig. S11. The stable SEI formation on our 3D electrode, accompanying the preservation of interior SnO_2_ coating layer, should be important for the stable cycling performance, as well as the highly reversible rate capacity.

To gain additional insight of the stable SnO_2_ coating layer, the voltage profiles of SnO_2_/Cu foam and SnO_2_ NPs were examined (Supplementary Fig. S12a,b). It is observed that the different electrochemical behavior during charge process especially at high voltages approximately over 1.0 V between the electrodes. Some portion of Li_2_O matrix decomposes upon delithiation above 1.0 V and oxygen resulted from the decomposition reacts with Sn for forming the SnO_x_^44^,^54^. From the comparison of the differential capacity profiles in charge process between the 2nd and 10th cycles of the SnO_2_/Cu foam and SnO_2_ NPs (Supplementary Fig. S12c), the area corresponding to the decomposition of Li_2_O and the oxidation of Sn to SnO_2_, obtained from the integrated charge in the differential curves at voltages higher than 1.0 V, decreases in both SnO_2_/Cu foam and SnO_2_ NPs after 10 cycles. In Supplementary Fig. S12d, the area for the partially reversible oxidation of Sn in SnO_2_ NPs decreases sharply after 30 cycles with a negligible amount of the decomposed Li_2_O, whereas that in SnO_2_/Cu foam is maintained considerably until the 50th cycle, suggesting that the SnO_2_ coating layer in Cu foam is geometrically stable.

In conclusion, we have developed a SnO_2_-coated 3D Cu foam by employing a facile and scalable combination of freeze-casting and sol-gel coating processes. The coated Cu scaffold with a 3D macroporous metallic network structure is fabricated and used in a lithium-ion battery anode for the first time. The unique 3D structure with a dual pore-size and pore-shape distribution enables fast transport of Li ion and electron, and accommodates the large volume changes of SnO_2_ coating layer during cycling. By taking these advantages of the Cu foam, we successfully achieved highly reversible capacity and superior rate capability, being close to the best performance of Sn-based nanoscale material reported to date. In addition, the foam fabrication by freeze-casting is versatile and can be extended to other metals than Cu and the foams can be applied to other high-capacity anode materials that currently experience cycling damage due to the large volume changes with repeated Li ion insertion and extraction.

## Methods

### Synthesis of copper layer-by-layer assembly via freeze-casting

Nano-sized cupric oxide (CuO) powder, with a particle size of 40–80 nm and 99.9% purity, was purchased from Inframat Advanced Materials (Manchester, CT., USA). Cupric oxide powder slurry was prepared through the following steps: 49.4 wt% cupric oxide powder and 2.5 wt% polyvinyl alcohol (PVA, Sigma-Aldrich Co., USA) binder were slowly suspended and dissolved in 30 mL deionized water, respectively, by using stirring and sonication. The slurry was then cooled to a few degrees above the freezing point of water and poured into a Teflon mold (54 mm in interior diameter, 77 mm in length) placed on a copper rod. The freeze-casting apparatus is shown schematically in Supplementary Fig. S13. The insulated steel container was filled with liquid nitrogen (outer) and ethyl alcohol (interior) and the temperature at the top of the copper rod was fixed at −10 °C using a heater. During directional freezing of the cupric oxide slurry, growth of vertical ice crystal colonies occurred, accompanied by entrapment of copper oxide particles between the ice crystals. Once freezing was completed, the sample underwent sublimation in a freeze dryer for 40 h at −88 °C under 0.005 torr vacuum. After sublimation of the ice, a continuous, macroporous, layered-structure resulted, consisting of elongated channels separated by interconnected, parallel walls consisting of lightly-bound copper oxide particles. The green-body foam was then reduced from copper oxide to copper in a hydrogen atmosphere. Reduction and sintering consisted of presintering at 250 °C for 4 h and primary sintering at 800 °C for 14 h; these were conducted in a tube furnace containing a 5% H_2_/Ar gas at a heating rate of 5 °C min^−1^. As shown in Supplementary Fig. S14a, the dark green CuO foam turned to a typical Cu orange after reduction in the hydrogen atmosphere and underwent a volume shrinkage of 44%. Prior to applying the SnO_2_ coating to the freeze-cast Cu foam, the Cu foam body was cut into thin coupons and polished to 300 μm thickness with colloidal alumina following standard metallographic procedures. The freeze-cast Cu foam with 300 μm thickness was employed to an electrode, but the thickness of Cu foam was much thicker than the conventional Cu foil current collector with 20 μm and also the previous 3D scaffold electrode with 160 μm^55^, 59 μm^56^, and 10 μm^20^. The large difference of the electrode height in magnitude has a chance to be more reduced by introducing a physically cutting or a compression process under high pressure^55^,^56^. Therefore, the possibility to improve the volumetric energy density by reducing the thickness of Cu foam can exist in this work. Finally, disks 11 mm in diameter were then punched out. The prepared Cu foam was heated at 500 °C for 1 h in a 5% H_2_/Ar mixture gas to burn off any remaining binder or leftover solution from the polishing process. Supplementary Fig. S14b indicates the development of Cu struts depending on the position of Cu foam body as indicated at top and middle positions. To obtain a dense scaffold, the layer at middle position was selected and prepared for the following cutting and polishing processes.

### SnO_2_ coating through a sol-gel process

A SnO_2_-based solution was prepared by dissolving 0.338 g of SnCl_2_·2 H_2_O in a solvent mixture consisting of 0.03 mL 37% hydrochloric acid and 0.47 mL ethanol to obtain a 3 M Sn(II) solution (0.564 g and 1.128 g of SnCl_2_·2H_2_O for 5 M and 10 M, respectively), which was subsequently aged at room temperature for 24 h. Triple deionized water (0.03 mL) was then added to the solution, which was aged for another 24 h. The Cu foam electrode was immersed in the prepared gel for 24 h, followed by solvent evaporation at 80 °C in vacuum. Heat treatment at 500 °C in an Ar atmosphere for 2 h converted the tin oxide precursor gel into crystalline SnO_2_ (Supplementary Fig. S15). The mass of coated SnO_2_ before and after sol-gel coating was weighed by a microbalance with an accuracy of 0.01 mg. The average SnO_2_ loading mass of electrode was 1.71 mg cm^−2^.

### Electrochemical measurements and characterization

The SnO_2_/Cu foam, without the addition of any binder or conductive agent, was assembled into a coin cell to be used as a working electrode, and was compared with a SnO_2_ powder electrode as control. For the control SnO_2_ powder electrode, commercial SnO_2_ NPs (<100 nm, purchased from Aldrich) or SnO_2_ powder directly obtained from sol-gel process was mixed with Ketchen Black as a conductive agent, and PVDF as a binder, in N-methyl-2-pyrrolidone (NMP) solvent. The SnO_2_:Ketchen Black:PVDF weight ratio was 70:15:15. The mixed slurry was uniformly pasted using a doctor blade method onto a Cu foil to serve as a conventional current collector. The electrode was dried under vacuum at 120 °C for 8 h. The average SnO_2_ loading mass of powder electrode was 1.82 mg cm^−2^. 2032-type coin cells, consisting of SnO_2_/Cu foam or SnO_2_ NPs as the working electrode and with a lithium metal foil as both the counter and the reference electrodes, were assembled in a glove box under a dry Ar atmosphere. The electrolyte used in this study was 1.0 M LiPF_6_ dissolved in a mixture of ethylene carbonate (EC) and diethyl carbonate (DEC) in a volume ratio of 1:1. Galvanostatic tests (WBCS3000 cycler, WonATech, Korea) were carried out on the coin cells at current rate of 1 C (781 mA g^−1^) in the voltage range of 2.0 V to 0.01 V (*vs.* Li^+^/Li) at 25 °C. XRD patterns were obtained with a Bruker D-5005 using Cu-Kα radiation (λ = 1.5406 Å), operating at 40 kV and 40 mA with a scan range of 20–80°. The morphologies of specimens were characterized using field emission scanning electron microscopy (FE-SEM), (Carl Zeiss, SUPRA 55VP). Pore size distribution and porosity of the Cu foam were analyzed using Mercury intrusion porosimetry (MIP), (AutoPore IV 9510, Micromeritics). X-ray photoelectron spectrometer (XPS) was operated on the surface of electrode with Ar ion beam etching during 120 s using VG Scientifics (Al Kα source). The results were calibrated by referencing C1s at 285 eV using Avantage software. Electrochemical impedance spectroscopy (EIS) was conducted at a charged state (2.0 V *vs.* Li^+^/Li) after the selected cycles in 10 mV amplitude with the frequency range from 100 kHz to 10 mHz (Zahner, Germany).

## Additional Information

**How to cite this article**: Um, J. H. *et al.* 3D macroporous electrode and high-performance in lithium-ion batteries using SnO_2_ coated on Cu foam. *Sci. Rep.* **6**, 18626; doi: 10.1038/srep18626 (2016).

## Supplementary Material

Supplementary Information
