# Supplementary material for: 3D macroporous electrode and high-performance in lithium-ion batteries using SnO2 coated on Cu foam
Source: Sci Rep. 2016 Jan 4;6:18626. doi: 10.1038/srep18626 (PMC4698716; doi:10.1038/srep18626)
Supplement: Supplementary Information [file srep18626-s1.pdf]

**Supplementary Information for**

# 3D macroporous electrode and high-performance in lithium-ion batteries using $SnO_2$ coated on Cu foam

Ji Hyun Um[1,2], Myounggeun Choi[3], Hyeji Park[3], Yong-Hun Cho[4], David C. Dunand[5], Heeman Choe[3] & Yung-Eun Sung[1,2]

[1]School of Chemical and Biological Engineering, Seoul National University, Seoul 151-742, Republic of Korea

[2]Center for Nanoparticle Research, Institute for Basic Science (IBS), Seoul 151-742, Republic of Korea

[3]School of Advanced Materials Engineering, Kookmin University, Seoul 136-702, Republic of Korea

[4]Department of Chemical Engineering, Kangwon National University, Samcheok 245-711, Republic of Korea

[5]Department of Materials Science and Engineering, Northwestern University, Evanston, IL 60208, USA

Correspondence and requests for materials should be addressed to Y.-H. C. (email: yhun00@ kangwon.ac.kr) or to Y.-E. S. (email: ysung@snu.ac.kr)

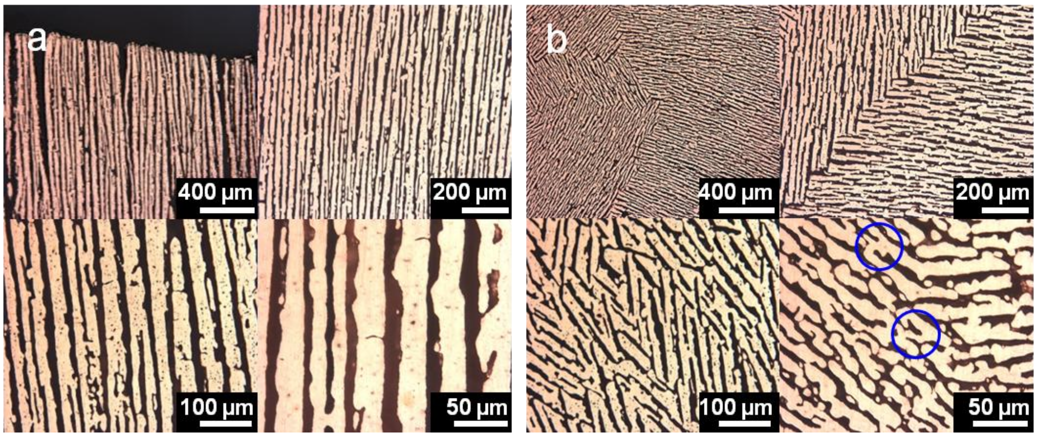

**Supplementary Figure S1** Optical microscopic images of a polished Cu foam, after being cut either (a) vertically or (b) horizontally. The blue circles indicate lamellar bridges crossing the gaps between adjacent lamellae.

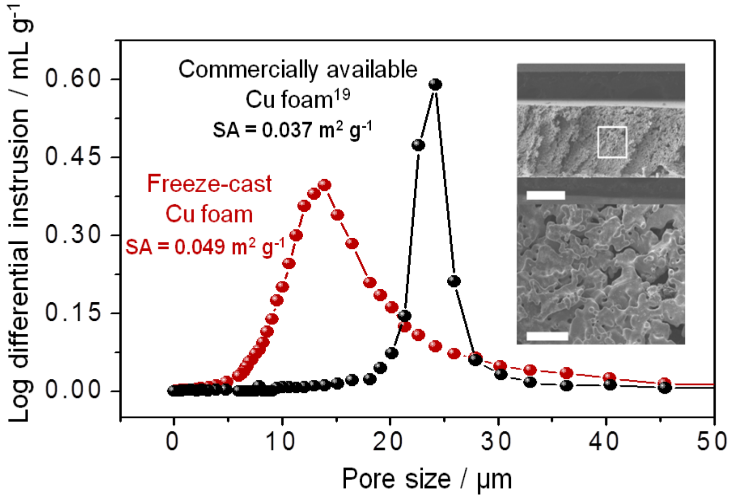


**Supplementary Figure S2** Pore size distributions of commercially available Cu foam applied to previous our work and freeze-cast Cu foam used in this work. The inset shows a cross-sectional SEM image of Cu foam via freeze-casting (top) and an enlarged SEM image of the region indicated by white rectangle in top image (bottom). Scale bars, 400 µm and 40 µm.

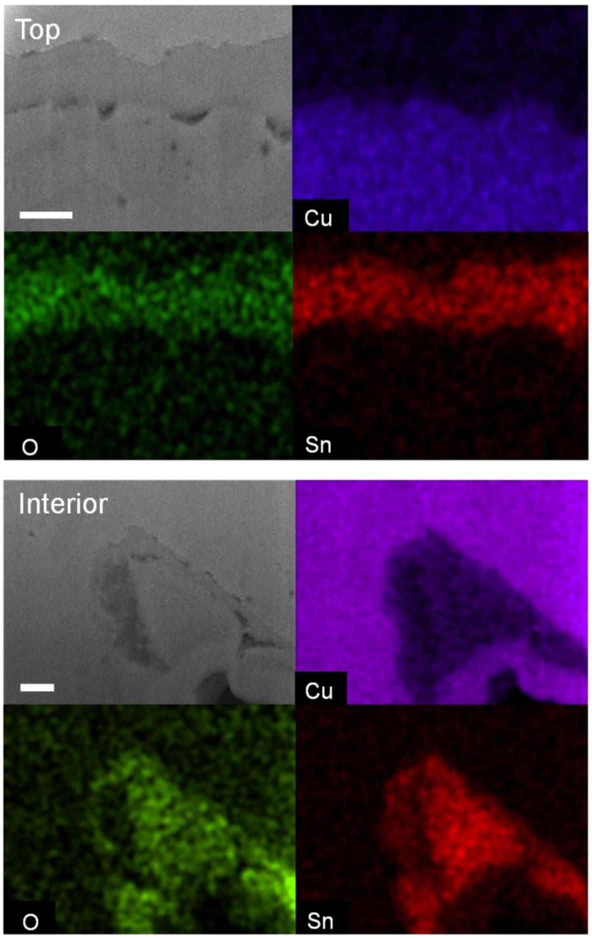


**Supplementary Figure S3** Cross-sectional SEM and EDX images of $SnO_2$/Cu foam at top and interior regions with element mapping of Cu, O, and Sn, respectively. Scale bars, 500 nm (top) and 500 nm (interior).

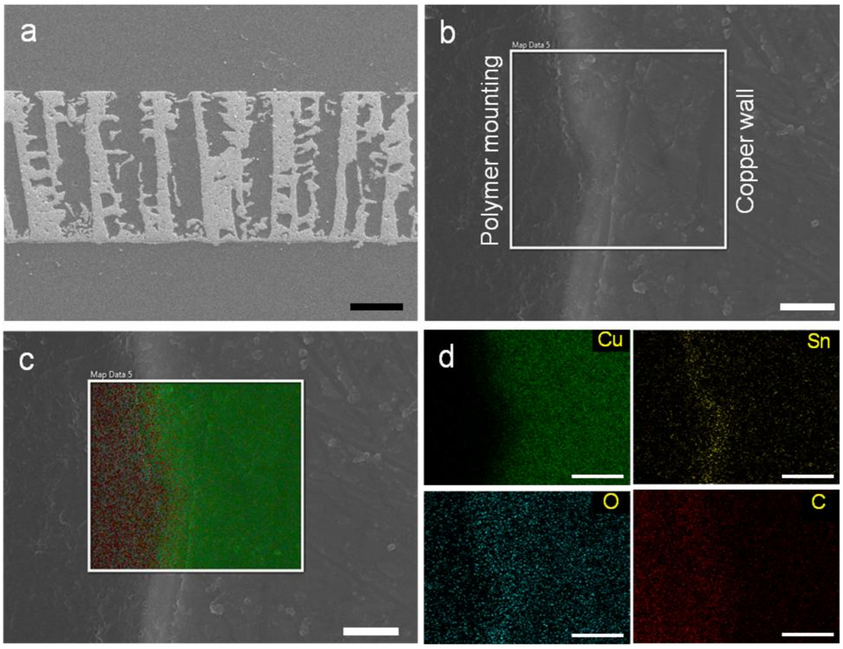


**Supplementary Figure S4** (a) An entire cross-sectional SEM image of $SnO_2$/Cu foam. Scale bar, 100 µm. (b) SEM image of the magnified surface on lamella in $SnO_2$/Cu foam interior. Scale bar, 500 µm. (c) EDX mapping image of (b). Scale bar, 500 µm. (d) Element distribution of Cu, Sn, O, and C, respectively. Scale bar, 500 µm.

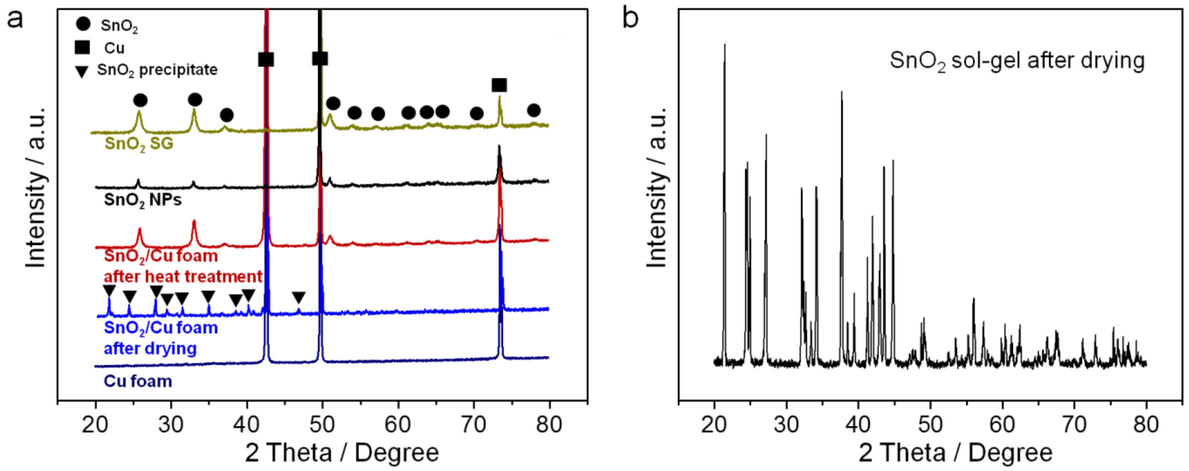


**Supplementary Figure S5** (a) XRD patterns of pristine Cu foam, $SnO_2$/Cu foam after drying and heat treat ment, $SnO_2$ NPs, and $SnO_2$ SG. (b) XRD pattern of $SnO_2$ sol-gel precipitate. There are no oxidation peaks such as CuO and $Cu_2O$ in the dried $SnO_2$/Cu foam. Only Cu and $SnO_2$ precipitate peaks resulted from the gelation and dehydration of $SnO_2$ sol are observed.

## Details on stability evaluation in oxidation of the 3D Cu foam

In both electrodes of the dried $SnO_2$/Cu foam and the annealed $SnO_2$/Cu foam, the copper oxides (CuO and $Cu_2O$) being possibly formed through wet synthesis route such as sol-gel, were not observed from XRD analysis as shown in Supplementary Fig. S5. However, due to the detection limitation of XRD analysis, trace of the CuO and $Cu_2O$ could be developed in the $SnO_2$/Cu foam electrode. Therefore, XPS analysis was conducted to examine the surface of Cu foam in the annealed $SnO_2$/Cu foam as the final product.

I. CuO

The XPS profile of annealed $SnO_2$/Cu foam is presented in Supplementary Fig. S5c. The two peaks located at around 934.9 eV and 954.8 eV are assigned to the binding energy of Cu $2p_{3/2}$ and Cu $2p_{1/2}$, respectively, which indicates the presence of $Cu^{2+}$ (CuO) in the $SnO_2$/Cu foam. In addition, the shake-up satellite peak at a binding energy approximately 9 eV higher than that of Cu$2p_{3/2}$ further confirms the existence of the $Cu^{2+}$ on the surface of $SnO_2$/Cu foam[R1,2]. XPS depth profiles with Ar ion beam etching were used to further probe the chemical oxidation state of $SnO_2$/Cu foam. The surface oxide layer of CuO is not detected after Ar ion etching after from 10 s to 120 s, confirming that the Cu foam maintains the metallic character under the thin surface oxide layer after the $SnO_2$ sol-gel coating.

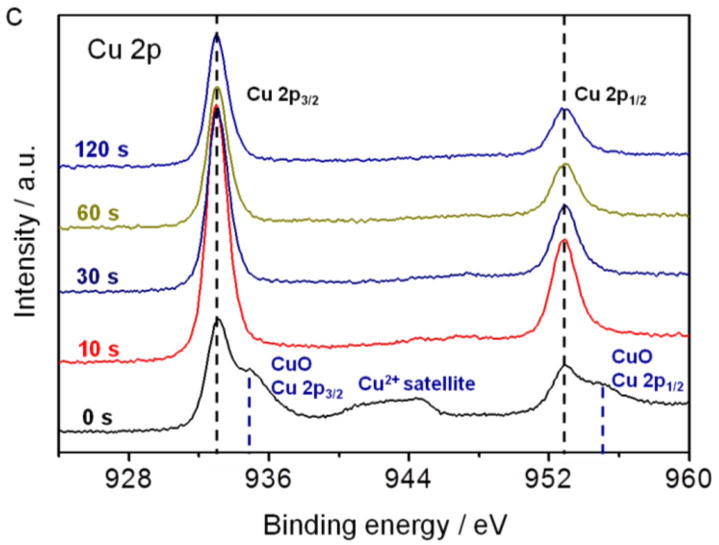


**Supplementary Figure S5** (c) XPS profiles of $SnO_2$/Cu foam with various Ar ion etching time.

By using the XPS analysis operating condition (240 keV, 1 μA, and 2 x 2 $mm^2$) and the below equation, the etching rate could be estimated[R3,4].

$$\frac{z}{t} = \frac{M_w}{\rho\, n_A e} S j_P$$

$M_w$ = Molar weight of the target [g/mol]
ρ = Density of material [g/$cm^3$]
$n_A$ = Avogadro number [$mol^{-1}$]
e = Electron charge [A·s]
S = Sputtering yield
$j_P$ = Primary ion current density [A/$cm^2$]

$$\frac{z}{t} = \left(\frac{63.5\ \mathrm{g}}{\mathrm{mol}}\right) \mathrm{x} \left(\frac{\mathrm{cm}^3}{8.96\ \mathrm{g}}\right) \mathrm{x} \left(\frac{\mathrm{mol}}{6.02\ \mathrm{x}\ 10^{23}}\right) \mathrm{x} \left(\frac{1}{1.6\ \mathrm{x}\ 10^{-19}\ \mathrm{A \cdot s}}\right) \mathrm{x}\ (6)^{*}\ \mathrm{x} \left(\frac{0.25\ \mathrm{x}\ 10^{-4}\ \mathrm{A}}{\mathrm{cm}^2}\right)$$

* The sputtering yield of copper at 240 keV can be estimated about 6 from the paper[R5].

The etching rate is calculated at 1.104 Å/s
And, the thickness of CuO during 10 s is about 1.1 nm.

The volume of CuO on the Cu foam can be estimated by using the surface area of Cu foam** and the thickness of CuO layer.
** The surface area could be calculated the specific surface area and the mass of Cu foam.

$$V_{\mathrm{CuO}} = A_{\mathrm{Cu\ foam}}\ \mathrm{x}\ z_{\mathrm{CuO}} = \left(\frac{0.049\ \mathrm{m}^2}{\mathrm{g}}\right)\ \mathrm{x}\ (0.07554\ \mathrm{g})\ \mathrm{x}\ (1.1\ \mathrm{x}\ 10^{-9}\ \mathrm{m})$$

From the $0.00407 \times 10^{-9}$ $m^3$ of $V_{CuO}$ and the 6.315 $g/cm^3$ of $\rho_{CuO}$, the mass of CuO on Cu foam is calculated at 0.0000257 g in the $SnO_2$/Cu foam electrode.

II. $Cu_2O$

In the case of $Cu_2O$, distinguishing it from metallic Cu in same XPS pattern is not easy due to the negligible difference of 0.2 eV between $Cu2p_{3/2}$ binding energies of $Cu_2O$ and Cu[R6].

Although the quantitative analysis of $Cu_2O$ through XPS analysis is difficult, by using XRD detection limitation (2~3 wt% with laboratory X-ray source and 0.1 wt% with synchrotron radiation source)[R7], approximately 3 wt% of the sample not observed in XRD pattern may be exist to maximum value in the $SnO_2$/Cu foam. When the mass of sample for XRD analysis is 0.002336 g, about 0.0000701 g of $Cu_2O$ is in the $SnO_2$/Cu foam electrode.

The mass contribution of CuO and $Cu_2O$ in total active material is approximately 2 wt% and 5 wt%, respectively.

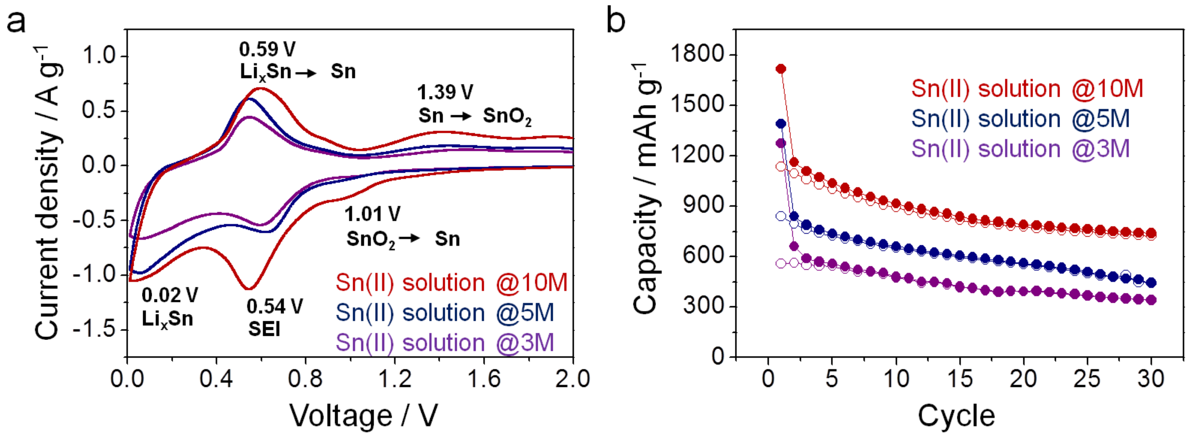


**Supplementary Figure S6** (a) Cyclic voltammograms of $SnO_2$/Cu foam obtained from different sol concentration at a scan rate of 0.1 mV $s^{-1}$. (b) Cycle performance at current rate of 1 C.

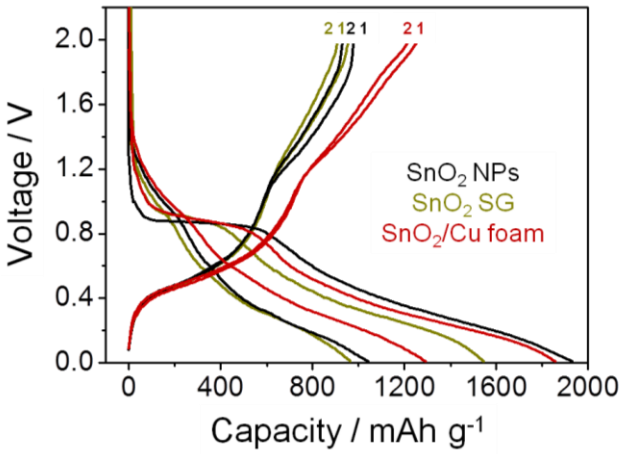


**Supplementary Figure S7** Voltage profiles of $SnO_2$/Cu foam, $SnO_2$ SGP, and $SnO_2$ NPs during the first two cycles at 0.5 C.

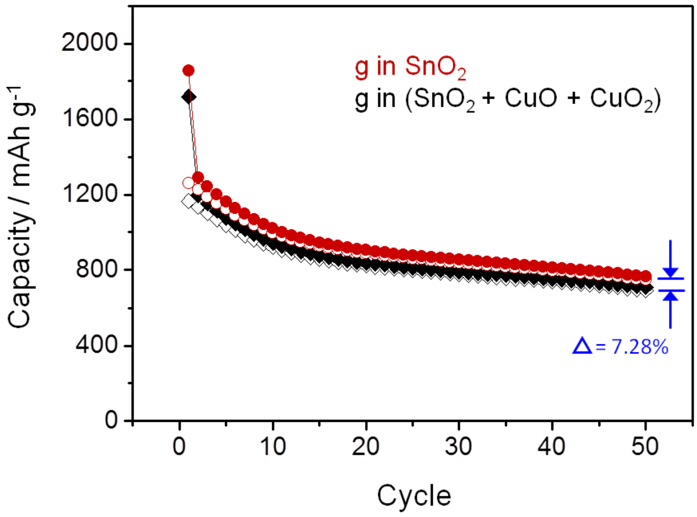


**Supplementary Figure S8** Performance comparison of $SnO_2$/Cu foam with consideration of surface oxidation of Cu foam.

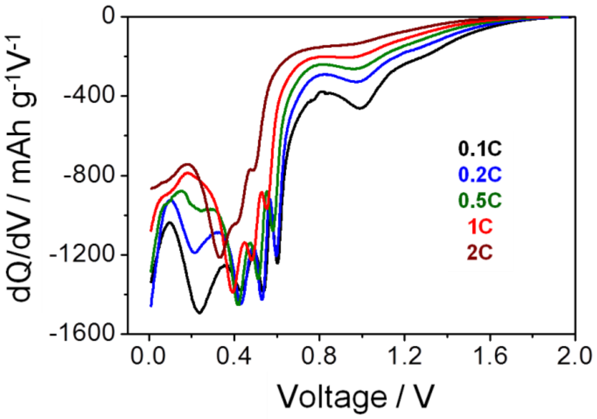


**Supplementary Figure S9** Differential capacity profiles of $SnO_2$/Cu foam differentiated from the final discharge voltage profile (Fig. 4d) in each current rate step.

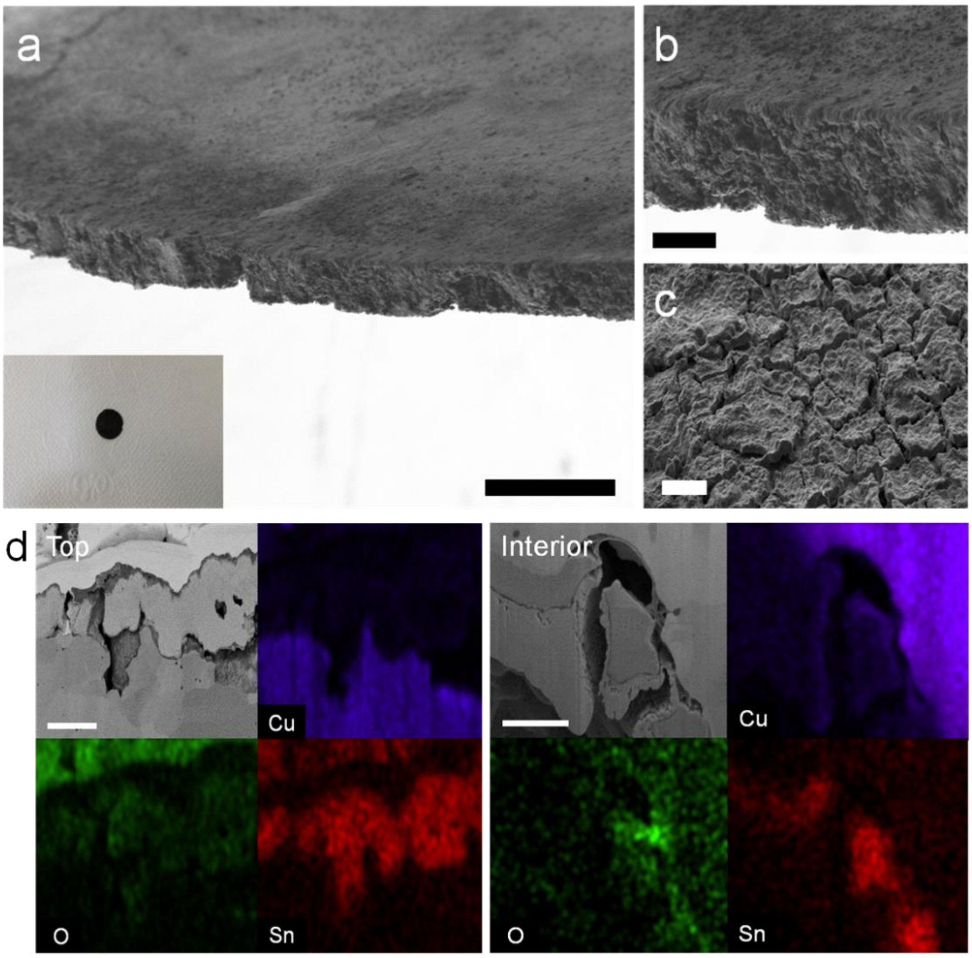


**Supplementary Figure S10** (a) SEM image of $SnO_2$/Cu foam after 50 cycles at 0.5 C. Scale bar, 400 μm. The inset shows the photograph of disassembled $SnO_2$/Cu foam electrode. (b) Side view and (c) Top view SEM images of the $SnO_2$/Cu foam. Scale bars, 80 μm (Side view) and 8 μm (Top view). (d) Cross-sectional SEM and EDX images of $SnO_2$/Cu foam after 50 cycles at 1 C at top and interior regions with element mapping of Cu, O, and Sn, respectively. Scale bars, 2 μm (top) and 1 μm (interior).

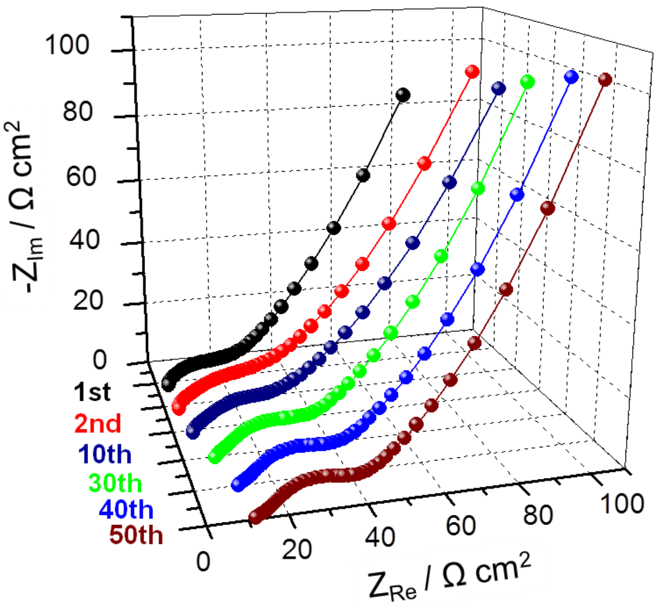


**Supplementary Figure S11** Cell impedance tests of $SnO_2$/Cu foam after the selected cycles at 1 C.

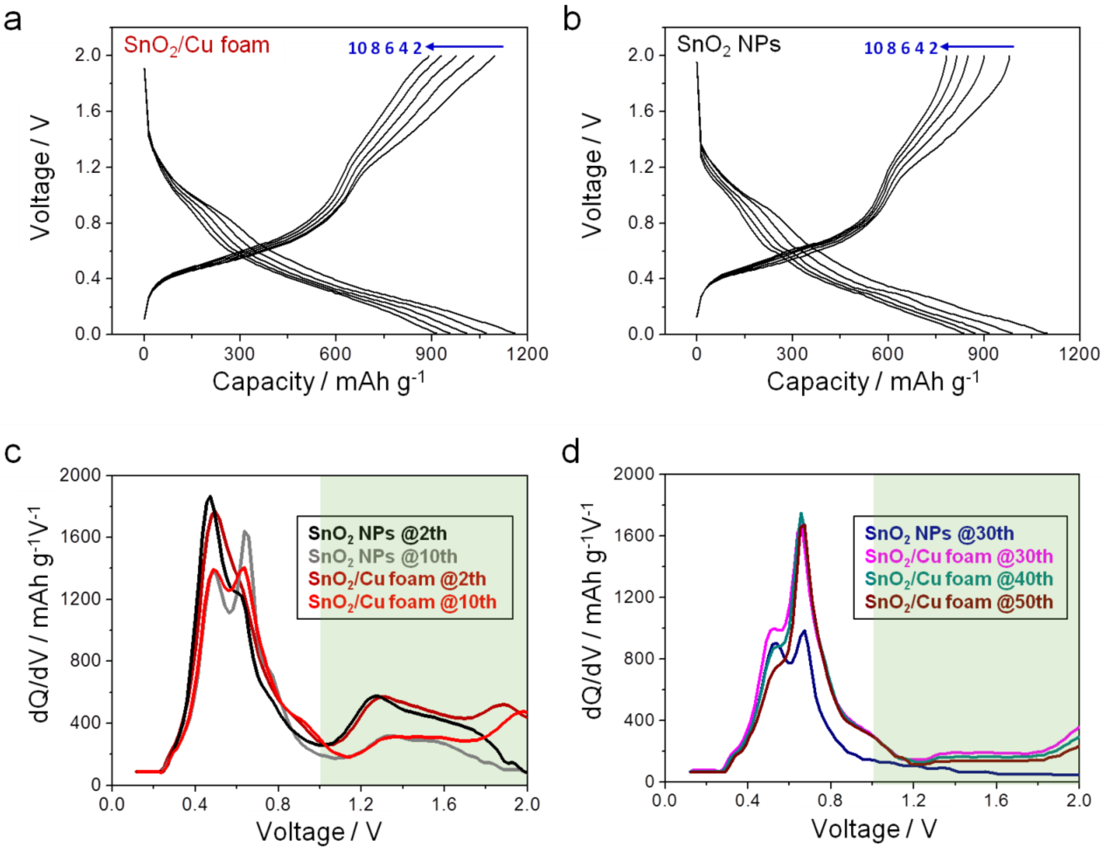


**Supplementary Figure S12** Voltage profiles of (a) $SnO_2$/Cu foam and (b) $SnO_2$ NPs after the selected cycles at 1 C. Differential capacity profiles of the $SnO_2$/Cu foam and $SnO_2$ NPs differentiated from the charge voltage profiles in (c) the initial period (2th and 10th cycles) and (d) the late period (30th, 40th, and 50th cycles).

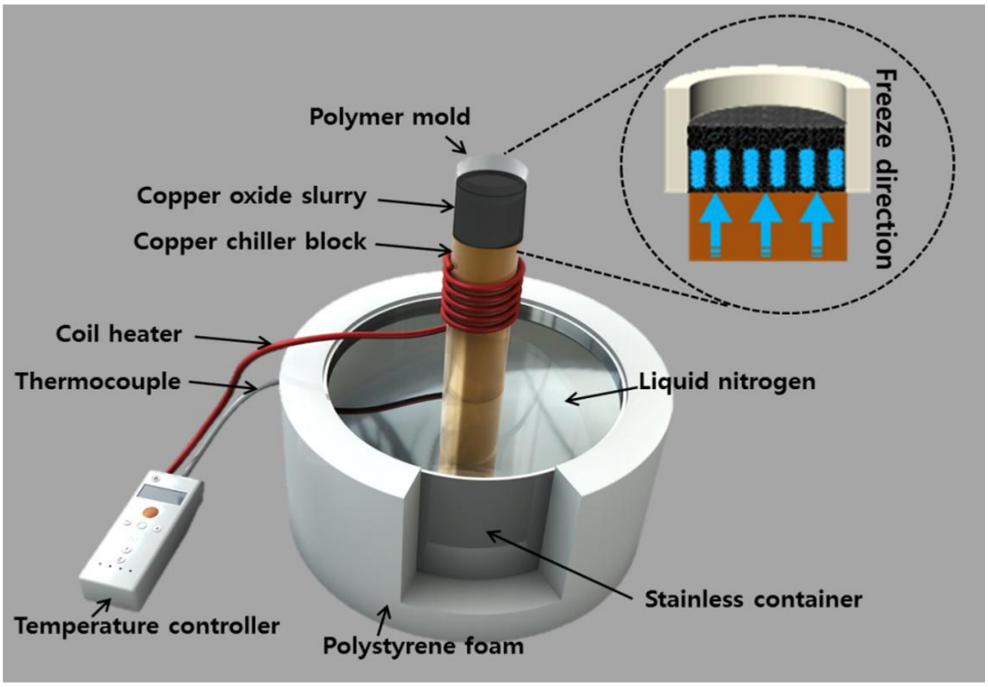


**Supplementary Figure S13** Schematic diagram of a freeze-casting apparatus.

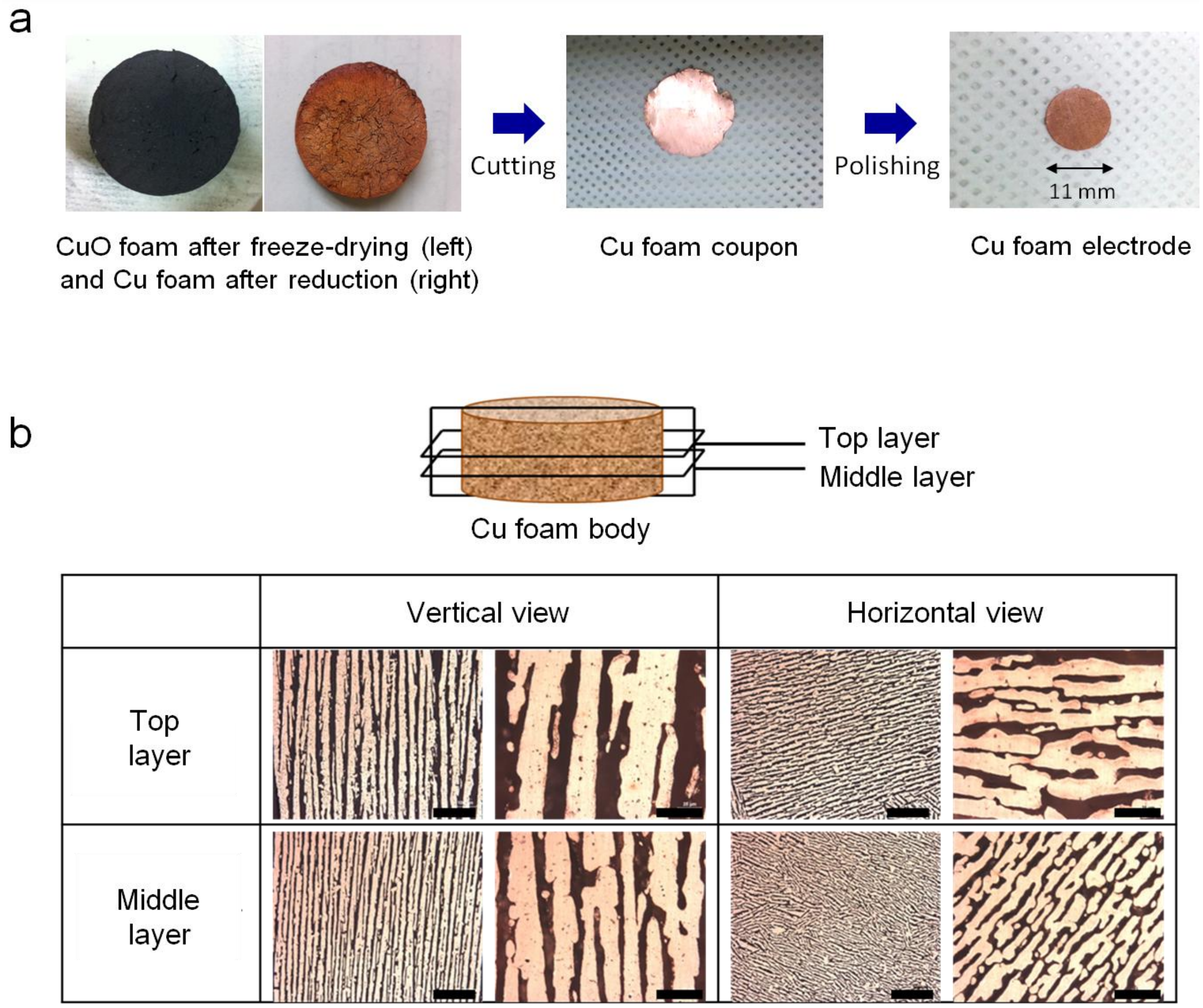


**Supplementary Figure S14** (a) Photographs of manufacturing process from CuO foam body to Cu foam electrode. (b) Optical microscopic images of the Cu foam electrode at top and middle positions. Scale bars, 200 µm (left) and 50 µm (right) in each individual space of a table.

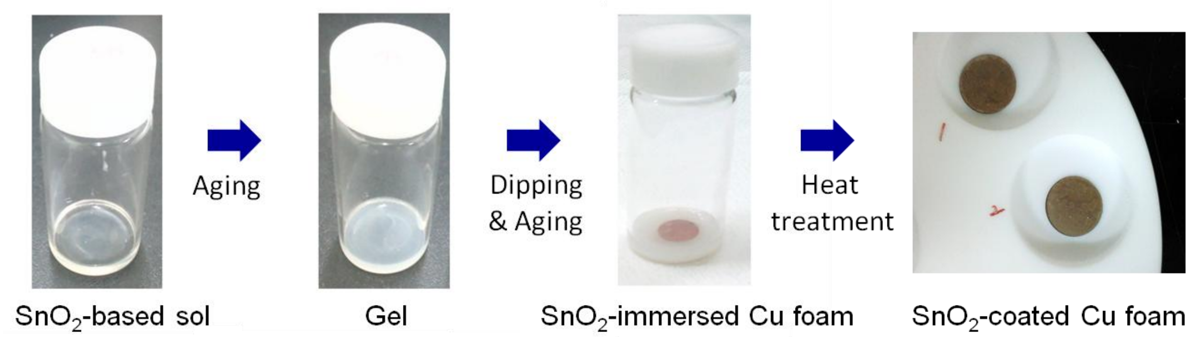


**Supplementary Figure S15** Photographs of $SnO_2$ sol-gel coating process.

R1. Yin, M. *et al.* Copper Oxide Nanocrystals. *J. Am. Chem. Soc.* **127**, 9506-9511 (2005).

R2. Sakai, Y., Ninomiya, S. & Hiraoka, K. XPS depth analysis of CuO by electrospray droplet impact. *Surf. Interface Anal.* **44**, 938–941 (2012).

R3. Smentkowski, V. S. Trends in sputtering. *Progress in Surf. Sci.* **64**, 1-58 (2000).

R4. Behrisch, R. & Wittmaack, K. *et al.* Sputtering by Particle Bombardment I, II and III. Springer Series 560, Topics in Applied Physics, Vol 47, 52 and 64 (Springer-Verlag Berlin Heidelberg, 1981, 1983 and 1991).

R5. Sigmund, P. Sputtering Yield of Amorphous and Polycrystalline Targets. *Phys. Rev.* **184**, 383–416 (1969).

R6. Poulston, S., Marlet, P. M., Stone, P. & Bowker, M. Surface Oxidation and Reduction of CuO and $Cu_2O$ Studied Using XPS and XAES. *Surf. Interface Anal.* **24**, 811-820 (1996).

R7. Drowley, C. I. 1-Application of Materials Characterization Techniques to Silicon Epitaxial Growth (ed. Strausser, Y.) 30 (Butterworth Heinemann, 1993).
